# Acceptability and Effectiveness of Artificial Intelligence Therapy for Anxiety and Depression (Youper): Longitudinal Observational Study

**DOI:** 10.2196/26771

**Published:** 2021-06-22

**Authors:** Ashish Mehta, Andrea Nicole Niles, Jose Hamilton Vargas, Thiago Marafon, Diego Dotta Couto, James Jonathan Gross

**Affiliations:** 1 Department of Psychology, Stanford University Stanford, CA United States; 2 Youper, Inc San Francisco, CA United States

**Keywords:** digital mental health treatment, acceptability, effectiveness, anxiety, depression

## Abstract

**Background:**

Youper is a widely used, commercially available mobile app that uses artificial intelligence therapy for the treatment of anxiety and depression.

**Objective:**

Our study examined the acceptability and effectiveness of Youper. Further, we tested the cumulative regulation hypothesis, which posits that cumulative emotion regulation successes with repeated intervention engagement will predict longer-term anxiety and depression symptom reduction.

**Methods:**

We examined data from paying Youper users (N=4517) who allowed their data to be used for research. To characterize the acceptability of Youper, we asked users to rate the app on a 5-star scale and measured retention statistics for users’ first 4 weeks of subscription. To examine effectiveness, we examined longitudinal measures of anxiety and depression symptoms. To test the cumulative regulation hypothesis, we used the proportion of successful emotion regulation attempts to predict symptom reduction.

**Results:**

Youper users rated the app highly (mean 4.36 stars, SD 0.84), and 42.66% (1927/4517) of users were retained by week 4. Symptoms decreased in the first 2 weeks of app use (anxiety: *d*=0.57; depression: *d*=0.46). Anxiety improvements were maintained in the subsequent 2 weeks, but depression symptoms increased slightly with a very small effect size (*d*=0.05). A higher proportion of successful emotion regulation attempts significantly predicted greater anxiety and depression symptom reduction.

**Conclusions:**

Youper is a low-cost, completely self-guided treatment that is accessible to users who may not otherwise access mental health care. Our findings demonstrate the acceptability and effectiveness of Youper as a treatment for anxiety and depression symptoms and support continued study of Youper in a randomized clinical trial.

## Introduction

Nearly half the people in the United States will have a mental disorder at some point during their life span [1,2], and many more will have subthreshold symptoms. The most frequent mental health conditions are anxiety and depression, jointly termed “emotional disorders,” and these impact 34% and 21% of people in the United States, respectively [3]. Despite the availability of effective treatments for emotional disorders, most people in need of treatment will not receive it [4]. Researchers have found that both structural (eg, financial, availability) and attitudinal barriers (eg, desire to handle problems independently) prevent patients from seeking mental health treatment [5]. Fully automated mental health intervention apps offer the promise of overcoming these barriers. By obviating the need for a trained clinician, the cost of treatment can be reduced by orders of magnitude and can be delivered to anyone with access to the internet. Moreover, fully automated treatments provide a means to treat patients who are uncomfortable seeking help from another person.

One particularly promising type of digital mental health intervention is a mobile app that can be installed on a person’s mobile device. The Apple and Google Play stores organize these apps, centralizing the location where interventions can be accessed and allowing users to vet apps by reviewing their descriptions in the store and reading user reviews. Once installed on a person’s phone, the mobile app medium makes it possible for people to access interventions anytime and anywhere. This opportunity has not been overlooked. Recent figures tally over 10,000 mental health apps available to consumers [6]. However, while the options for mental health treatment apps are at an all-time high, there is little research on the acceptability of available app-based treatments and whether they can actually reduce symptoms of psychopathology. In particular, mobile apps that are completely self-guided, and hence maximally accessible and scalable, are especially understudied. That said, a significant portion of the literature supports the efficacy of self-guided cognitive behavioral therapy administered via the internet, which patients may or may not be able to access on mobile phones depending on the intervention [7,8]. Thus, the potential for mobile apps to demonstrate similar efficacy is promising. The present study aimed to assess the acceptability and effectiveness of a self-guided intervention app called Youper that targets emotional disorders.

Although few in number, a handful of randomized controlled trials (RCTs) examining fully self-guided mental health intervention apps have shown promise. One mobile cognitive behavioral therapy intervention similar to Youper that employs a humanlike, chatbot interface found that college students experienced significantly reduced depression symptoms over the course of 2 weeks [9]. A number of RCTs have demonstrated the efficacy of self-guided mobile treatment programs for depression, including 1 testing problem-solving therapy and cognitive training [10], 3 testing cognitive behavioral therapy interventions [11-13], and 1 testing acceptance-based therapy [14]. Small-scale observational studies have also shown positive results for self-guided app-based treatments for symptoms of depression and anxiety [15,16].

Although these studies are promising, the evidence on self-guided digital mental health treatment is limited by small sample sizes obtained almost exclusively via RCTs. RCTs are considered the gold standard of evidence and play an indispensable role in assessing the efficacy of medical interventions. However, real-world evidence provides a necessary complement to understanding the impact of an intervention in the context that it will be received [17]. This fact has been acknowledged by numerous government bodies including the National Institute of Health, the Food and Drug Administration, and the European Medicines Agency, who have called for real-world evidence on interventions [18-20]. This call arises from the recognition of external validity shortcomings in RCTs due to factors such as inclusion criteria and differences in treatment adherence [21-23]

First, RCTs may be composed of different populations than those that naturally seek digital mental health treatment. Participants in RCTs are acquired through recruiting efforts and are then selected based on specific inclusion criteria. For example, participants in RCTs may be required to meet a minimum threshold of depression symptoms [10]. In the real world, digital mental health treatment (often distributed via smartphone app stores) is available to anyone with a smartphone and users span the range of depression symptomatology levels. Thus, the distribution of potential app users may or may not match the distribution of participants seen in the small body of existing literature on self-guided digital mental health. Further, it is plausible that participants who enroll in clinical mental health trials are more comfortable with seeking external mental health care than are users that discreetly download an app on their phone. Since targeting populations who carry stigma against seeking mental health care is an important goal and potential advantage of this technology, it is important that the populations being studied have equivalent attitudes to the population of potential users.

Second, treatment adherence in RCTs may systematically differ from real-world app usage because of the different experience that a participant in a clinical trial has compared to a user who downloads an app. In clinical trials, participants are often paid money to participate. Paid participants may feel a social obligation to adhere to the treatment plan. However, in real life, where app-based treatment is a completely individual experience, it is unclear whether adherence will be equivalent. Moreover, RCTs require, at minimum, an initial contact with study coordinators and sometimes additional contacts throughout the study. As contact with a treatment provider is known to increase adherence, this initial contact could boost levels of engagement [24]. Because the degree to which one engages and adheres to a treatment is related to treatment success [7], it is critical that we supplement evidence gained from RCTs with an understanding of how treatment recipients organically experience the app in the real world.

We define artificial intelligence (AI) therapy as a digital and fully automated, mobile, psychological treatment program that uses a conversational interface to deliver just-in-time adaptive interventions. The 3 key features that set AI therapy apart from traditional digital intervention approaches are (1) the use of a conversational (chatbot) interface, (2) inclusion of just-in-time interventions, and (3) adaptation and personalization. A primary goal of this study is to test whether AI therapy has potential as a viable treatment approach.

An additional goal of this study is to test the theoretical model underlying the just-in-time approach, which is a critical feature of AI therapy. Although just-in-time approaches have been used to target health behaviors, such as alcohol use, smoking, and obesity, only a small number of studies have described this approach in relation to emotional disorders [25,26].

Just-in-time interventions are designed to help the user manage moment-to-moment challenges that accumulate to negatively impact broader mental health functioning and produce symptoms of psychiatric disorders. Thus, just-in-time interventions target a proximal outcome that is theorized to accumulate over time to impact a longer-term outcome. In the case of anxiety and depression symptoms, emotion dysregulation is theorized to be a proximal cause for the manifestation of symptoms as well as a target for intervention [27,28]. Consistent with this hypothesis, a prior study of a just-in-time intervention for depression showed that it had promising impacts on depressive symptoms, albeit in a sample with just 10 people [25]. Following this theoretical work, we hypothesized that users who repeatedly succeed in regulating negative emotions by engaging just-in-time digital mental health interventions will experience long-term symptom reduction through accumulation of these regulation successes. We call this the *cumulative regulation hypothesis*. In addition to assessing AI therapy’s effectiveness for symptom reduction, the current study will test the cumulative regulation hypothesis by testing the association between the accumulation of successful emotion regulation efforts with symptom reduction over time.

In this paper, we examine the acceptability and effectiveness of AI therapy as it is implemented in a smartphone app called Youper. Our study had 3 aims. First, we explored the acceptability of Youper by analyzing user ratings and retention metrics. Second, we examined effectiveness by measuring the reduction in anxiety and depression symptoms in the first month of app use. We hypothesized that users of Youper would experience a reduction in anxiety and depression symptoms during this time period. Third, we tested the cumulative regulation hypothesis by examining the longitudinal relationship between success in downregulating acute negative emotion in Youper conversations and clinical symptoms. We hypothesized that within-session emotion regulation success during Youper engagements would predict greater reductions in anxiety and depression symptoms. Finally, in exploratory analyses, we examined whether demographics, including gender and age; and clinical characteristics, including the number of self-reported diagnoses, current psychotropic medications, and concurrent therapy, could predict symptom reduction over time.

## Methods

### Participants

Participants were Youper subscribers (ie, users who paid for full access to Youper) who downloaded the app between March 4, 2020, and July 10, 2020. This time frame was selected because Youper was relatively stable during this period (ie, no significant updates or changes to the intervention were deployed during this time). Subscribers paid US $44.99 to have unlimited access to Youper’s interventions for 1 year. Users who did not subscribe were only able to access the emotion regulation interventions once as part of a free sample, and therefore, were not included in this analysis.

Of 5943 users who completed at least one symptom measure in the study timeframe, 76.01% (n=4517) agreed for their data to be used for research, leaving a useable sample of 4517 participants. The sample was composed of 81.62% women (n=3687), 14.15% men (n=639), and 3.43% nonbinary individuals (n=155), and the average age of participants was 28.73 years (SD 9.63). Additional participant demographics and clinical characteristics are presented in Table 1. Participants completed symptom assessments at baseline (T0; within 3 days of subscribing to Youper), 2 weeks after baseline (T1), and 4 weeks after baseline (T2). Assessments were available to users every 14 days, and the majority of users completed their assessments within 3 days of them becoming available. Participants received access to anxiety or depression symptom measures based on their responses to screening questions. Symptom measures were administered if they endorsed a history of being diagnosed with clinical anxiety or depression, or if they reported elevated anxiety or depression symptoms on 2-item screening measures. Participants could receive only an anxiety measure, only a depression measure, or both depending on their responses to the screening items. Throughout the course of the measurement period, participants engaged in emotion regulation interventions at their discretion when emotional episodes arose.

**Table 1:** Additional demographic and clinical characteristics (N=4517).

Variable		Value, n (%)
**Occupation**		
	Employed full time	2221 (49.17%)
	Work and attend school	708 (15.67%)
	Student full time	493 (10.91%)
	Unemployed	420 (9.30%)
	Freelancer or work part time	397 (8.79%)
	Homemaker	149 (3.30%)
	No response	93 (2.06%)
	Retired	36 (0.80%)
**Operating system**		
	iOS	4038 (89.40%)
	Android	479 (10.60%)
**Talked to a doctor about emotional health**		
	Yes	3559 (78.79%)
	No	954 (21.12%)
**Self-reported diagnoses^a,b^**		
	Anxiety disorder	2479 (54.88%)
	Depressive disorder	2412 (53.40%)
	Any diagnosis	2994 (66.28%)
**Current treatment type^c^**		
	Prescribed medication	1904 (42.15%)
	Psychotherapy	1196 (26.48%)
	Prescribed medication or psychotherapy	2161 (47.84 %)

^a^Users were only asked about diagnoses if they reported talking to a doctor about their emotional health (n=3559).

^b^Mean number of diagnoses=2.82 (SD 1.47).

^c^Users were only asked about treatment if they reported a diagnosis (n=2994).

### Youper Intervention

**Figure 1:**
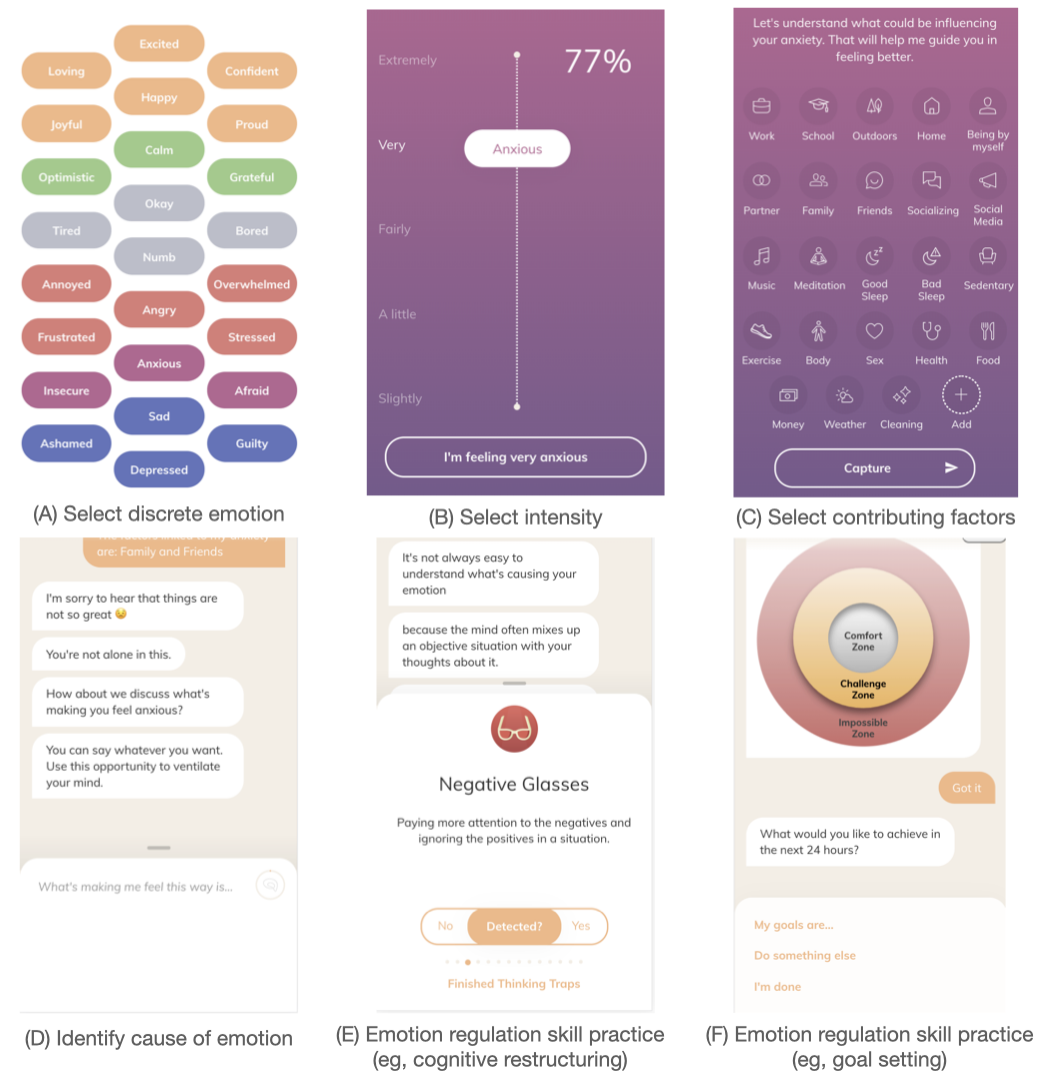
Example interaction with Youper. Users start by reporting a discrete emotion (A) and the intensity (B) which they feel the emotion. They then report which factors contributed to the emotion (C) and describe the precipitating event (D). Next, they proceed through a randomly selected intervention (eg, E or F) from the list (see Table 2). Finally, they report their discrete emotional state again and the intensity which they feel that emotion (A and B).

Youper is a novel intervention approach that aims to enhance the user’s emotion regulation skills using empirically supported treatments for anxiety and depression. Although the emotion regulation strategies employed in Youper have precedent in existing treatment protocols for anxiety and depression, the adaptation of these interventions to help a user manage emotional distress at the present moment is novel. Youper’s intervention is delivered via a conversational (ie, chat) interface and is entirely automated. Youper primarily uses a decision tree to select its responses to the user input. Each interaction with Youper is called a “conversation.” Conversations follow a prespecified sequence (see Figure 1 for examples): identify current emotion and intensity (0%-100%), select contributing factors from a prespecified list, complete an open text entry about what is causing the current mood, complete emotion regulation skill practice for a negative mood or wellness practice for a positive mood (see Table 2), and identify current emotion and intensity (0%-100%).

The goal of each conversation is to help the user learn adaptive emotion regulation skills. If the user is experiencing a negative emotion, the skill targets the current emotion. If the user is experiencing a positive emotion, the skill encourages upregulation of that emotional state. If the user is in a neutral state, the skills encourage practice of activities that promote emotionally adaptive behaviors and attentional and cognitive control. Youper primarily uses just-in-time interventions (delivered at the moment of need) to help users practice and learn skills for emotion regulation. Youper’s interventions target the 3 categories of treatment mechanisms defined by the common elements framework [29].

The common elements framework provides a review of common elements across cognitive and behavioral therapies inclusive of both traditional (cognitive therapy, behavioral activation) and third-wave (acceptance and commitment therapy, dialectical behavior therapy) approaches. They identify 3 mechanistic targets common to multiple effective therapies, including attention change (improving attentional focus and flexibility), cognitive change (improving ability to change perspective on an event), and context engagement (engaging new internal and external contexts to counteract maladaptive patterns). Youper’s interventions aim to increase emotion regulation skills by targeting these common elements. For example, Youper includes interventions to increase attentional control such as mindfulness, cognitive change such as cognitive restructuring or gratitude journaling, and context engagement via behavioral activation exercises. The common elements framework was used to guide the development of Youper’s interventions due to the extensive empirical support for the efficacy of each of these targets in enhancing emotion regulation and reducing symptoms of emotional disorders [30-36].

Each intervention is described in Table 2. Each skill follows a series of steps modeled after existing treatment manuals or research protocols. Skills practice includes a variety of formats including open-text entry following a prompt, graphical user interfaces, written content delivered via the chat, and audio.

**Table 2:** Emotion regulation change process targets and interventions.

Change process with interventions		Description
**Context engagement**		
	Behavioral activation	1. Selecting a rewarding or social activity from a list 2. Psychoeducation about action/motivation cycle 3. Setting a goal to complete the activity
	Goal setting	1. Psychoeducation about setting challenging, specific goals 2. Identifying a goal 3. Setting a reminder to check about goal completion
	Problem solving	1. Identifying the problem 2. Identifying a goal 3. Brainstorming solutions 4. Selecting a solution and setting a goal
**Attention change**		
	Mindfulness	1. Selection from a list of audio-recorded mindfulness exercises such as following the breath, progressive muscle relaxation, and mindfulness of thoughts
	Sleep relaxation	1. Visualization of calming scenery 2. Selection from a list of different types of white noise
**Cognitive change**		
	Acceptance	1. Practicing accepting negative thoughts and feelings without trying to change them 2. Planning to engage in value-driven behavior
	Cognitive restructuring	1. Identifying thoughts 2. Identifying cognitive distortions 3. Examining evidence 4. Identifying alternative thoughts
	Gratitude journaling	1. Identification of things for which the user is grateful
	Self-compassion	1. Identification of how the user would treat a friend dealing with difficult emotions 2. Identification of how to treat oneself with the same compassion

### Measures

#### Acceptability Measures

##### User Ratings

To assess acceptability of the Youper intervention, we asked users to provide a rating of the app using a 5-star scale. Users were given the following prompt: “I’d love to know how our journey together is going so far.” Users then provided their rating of Youper by selecting a number of stars ranging from 1 to 5. Users then were asked to provide feedback using an open text box.

##### Retention

Retention was measured as the proportion of Youper subscribers who engaged with the app during week 1, 2, 3, and 4 after subscription, as well as the average number of conversations that users had during each of these weeks.

#### Anxiety and Depression Symptoms

Anxiety symptoms were measured using the 7-item generalized anxiety disorder measure (GAD-7) [37]. The GAD-7 is a widely used measure of generalized anxiety disorder symptom severity and is frequently used as a general measure of overall anxiety symptoms. It has demonstrated excellent psychometrics with a Cronbach α of .92 and a sensitivity and specificity of 89% and 82%, respectively, for classifying generalized anxiety [37]. Depression symptoms were measured using a modified version of the Patient Health Questionnaire-9 (PHQ-9) with the suicide-related item removed and 1 of the items divided into 2 separate items [38]. Specifically, the item that asks if the respondent “has been moving slowly or has been fidgety and restless” was divided into an item about “moving slowly” and another item about “being fidgety and restless.” The PHQ-9 is a widely used measure of depression symptom severity with excellent psychometrics. The PHQ-9 has a Cronbach α of .89 and has both a sensitivity and a specificity of 88% for classifying major depression. In our slightly modified PHQ, we observed a comparable Cronbach α of .84 (95% CI 0.83-0.85) indicating good reliability.

#### Predictors of Symptom Reduction

##### Within-Session Emotion Regulation

To test the cumulative regulation hypothesis, we derived a measure of cumulative emotion regulation success. At the beginning of each Youper conversation, users selected their current emotion from a list of possible emotions as well as the intensity of that emotion (see Figure 1). Users who selected a negative emotion were also asked to report their emotion at the end of the conversation with Youper. We classified cases where users started with a negative emotion and ended with either a positive emotion or with a less intense negative emotion as a within-session emotion regulation “success.” We classified cases where users reported a worsening or unchanging negative emotion as a “failure to regulate.” To calculate a measure of cumulative within-session regulation success, we computed the proportion of cases classified as a success out of all conversations that started with a negative emotion.

As discrete negative emotion words encode different emotional intensities, we scaled the numeric self-reported emotional intensity according to an intensity scale factor corresponding to the discrete emotion the user selected. To derive the intensity scale factor for each discrete emotion, we first obtained normative valence and arousal ratings from a database of words that have been rated on a scale of 1 to 9 by a large sample of participants [39]. Next, we subtracted a constant (C=6) from the normative valence ratings, chosen so that all negative valence words would have negative-valued ratings and positive valence words would have positive ratings. To compute the intensity scale factor for each emotion word, we took the square root of the sum of the squared valence and arousal ratings (ie, the L2 norm). This decision was premised on the assumption that emotion intensity is a composite of valence and arousal [40]. Finally, we multiplied the self-reported numeric intensity by the intensity scale factor for the given emotion to obtain a scaled emotion intensity rating that could be compared across discrete emotion categories.

The scaling procedure had the effect of incorporating both the intensity of the emotion word and the self-reported numeric intensity into a single value which could be used to assess emotion regulation success pre- to postintervention. For example, without scaling, a participant that went from a rating of “75 annoyed” to “70 angry” would be erroneously classified as an instance of successful downregulation of negative emotion, despite the higher intensity imbued in the word “angry.” With scaling, “75 annoyed” would translate to “–383” and “70 angry” would translate to “–482,” and the increase in magnitude of negative emotion would result in a classification of failure to regulate. However, if the participant went from “75 annoyed” to “30 angry,” the “30 angry” rating would be scaled to “–207,” and the instance would be classified as a regulatory success. This procedure allowed us to use both the text information and numeric information in our assessment of success or failure to regulate emotions. As a check of robustness, we ran all analyses without the scaling procedure, and the results were substantively similar. To be conservative, we ultimately dichotomized these scaled scores into regulation successes and failures because, despite appearing to have a continuous measure of emotion regulation success, we were not confident that these scores truly represented precise gradations along a continuum.

##### Demographic and Clinical Characteristics

We examined both demographic and clinical characteristics as predictors of symptom reduction. Demographic characteristics included age (continuous) and gender (multinomial; man, woman, and nonbinary). Clinical characteristics included number of self-reported diagnoses (continuous), whether the user was currently taking psychotropic medication (binary), and whether the user was currently receiving psychotherapy (binary).

### Statistical Analyses

#### Aim 1: Acceptability

We report descriptive statistics for user retention and app ratings.

#### Aim 2: Effectiveness

To estimate symptom reduction as a function of time, we fit piecewise multilevel models in R (version 4.0.2; The R Foundation for Statistical Computing) using the package “lmerTest” (version 3.1-2) [41]. Consistent with prior work, we selected a piecewise approach to capture a typical pattern of symptom reduction observed in treatment studies where symptoms initially decrease sharply and then level out as time progresses [42-47]. In these models, we regressed the symptom outcome measure (GAD-7 score or PHQ score) onto the number of days since subscribing to the app.

We selected multilevel models because our outcome measures were nested within individuals as a result of repeated measurement at multiple timepoints. Multilevel models allow for the estimation of within-subject effects. Further, when fit with maximum likelihood, multilevel models allow for the inclusion of participants with incomplete data without deletion or imputation and produces unbiased estimates for model parameters [48,49]. As per guidelines for randomized clinical trials, we conducted an intent-to-treat analysis, including all participants who had at least one assessment [50-53]. As discussed by Gupta [51], “intent-to-treat analysis avoids overoptimistic estimates of the efficacy of an intervention resulting from the removal of non-compliers by accepting that noncompliance and protocol deviations are likely to occur in actual clinical practice.” We estimated the reduction of symptoms from T0 to T1 and from T1 to T2. We used a breakpoint at 14 days, as participants’ second of 3 symptom measurements was available to be completed 14 days after the first measurement. Because not all participants completed assessments immediately when they were available, we chose to treat time as a continuous predictor in our analysis rather than simply grouping observations into time points at T0, T1, and T2. This approach allowed us to keep all information that we had about the time that had elapsed from baseline and was more conservative because it did not assume that a change occurring more than 14 days after baseline was occurring exactly at 14 days.

The models included 2 fixed effect parameters: one which estimated the slope of symptom reduction from the start of using Youper to 14 days later, and another which estimated the slope of symptom reduction from the 14-day mark onward. Additionally, we included a random intercept term for each participant. We calculated Cohen *d* effect sizes by dividing the mean difference in symptom levels by the square root of the sum of the participant-level intercept variance and the residual variance [54].

#### Aim 3: Cumulative Regulation Hypothesis

To test the cumulative regulation hypothesis (ie, whether cumulative emotion regulation success within conversations predicted subsequent psychopathology symptoms), we fit longitudinal path analysis models for each of the 2 symptom measures (GAD-7 and PHQ) in the R package, “lavaan” (version 0.6-6) [55]. We fit these models using full information maximum likelihood and allowed the covariances between exogenous variables to be freely estimated [56]. This method enabled us to conduct an intent-to-treat analysis, including all participants that had a measurement for at least one variable included in the model. In these models, we estimated all autoregressive paths and lagged paths from emotion regulation success to subsequent clinical symptoms. Specifically, each path analysis model consisted of 3 regression equations. In the first equation, we regressed the T1 symptom outcome onto the T0 symptom outcome and the proportion of within-session regulation successes between T0 and T1 (ie, the proportion of negative emotions that were successfully regulated of the total number of negative emotion regulation attempts). In the second equation, we regressed the proportion of within-session regulation successes between T1 and T2 onto the proportion of within-session regulation successes between T0 and T1. Finally, we regressed the T2 symptom outcome onto the T1 symptom outcome and the proportion of within-session regulation successes between T1 and T2. (See Figure 3 for an illustration of paths with standardized coefficients.)

#### Exploratory Analyses: Clinical and Demographic Predictors

To test predictors of treatment response, we fit piecewise mixed effects models like those used in Aim 2, with the addition of interaction terms for the specified predictor. Specifically, we regressed the symptom outcome onto the interaction of the specified predictor and the number of days since the participant subscribed to the app. We examined age (continuous), gender (dummy coded with female as the reference group), number of self-reported diagnoses (continuous), whether the user was taking psychotropic medication (binary), and a whether the user was in therapy (binary) as individual difference predictors of symptom reduction.

## Results

### Aim 1: Acceptability

#### User Ratings

On the 5-point star rating scale, the median Youper rating was 5 and the mean was 4.36 (SD 0.84). Out of 3667 users who rated the app, 56.09% (n=2057) gave a 5-star rating, 26.89% (n=986) gave 4 stars, 14.97% (n=549) gave 3 stars, 1.31% (n=48) gave 2 stars, and 0.74% (n=27) gave 1 star. The mean conversation number at which users provided a rating was conversation 1.40, and the SD was 4.03.

#### Retention

Of the 4517 users who subscribed to Youper between March 4, 2020, and July 10, 2020, 90.75% (n=4099) were still using Youper in week 1, 60.44% (n=2730) were using in week 2, 51.78% (n =2339) were using in week 3, and 42.66% (n=1927) were using in week 4 after subscription. The average numbers of conversations users had in weeks 1, 2, 3, and 4 were 6.50 (SD 6.74), 3.08 (SD 4.50), 2.36 (SD 3.98), and 2.04 (SD 3.83), respectively. Across the whole 4-week period, users engaged in an average of 13.98 conversations (SD 16.89).

### Aim 2: Effectiveness

#### Anxiety

**Figure 2:**
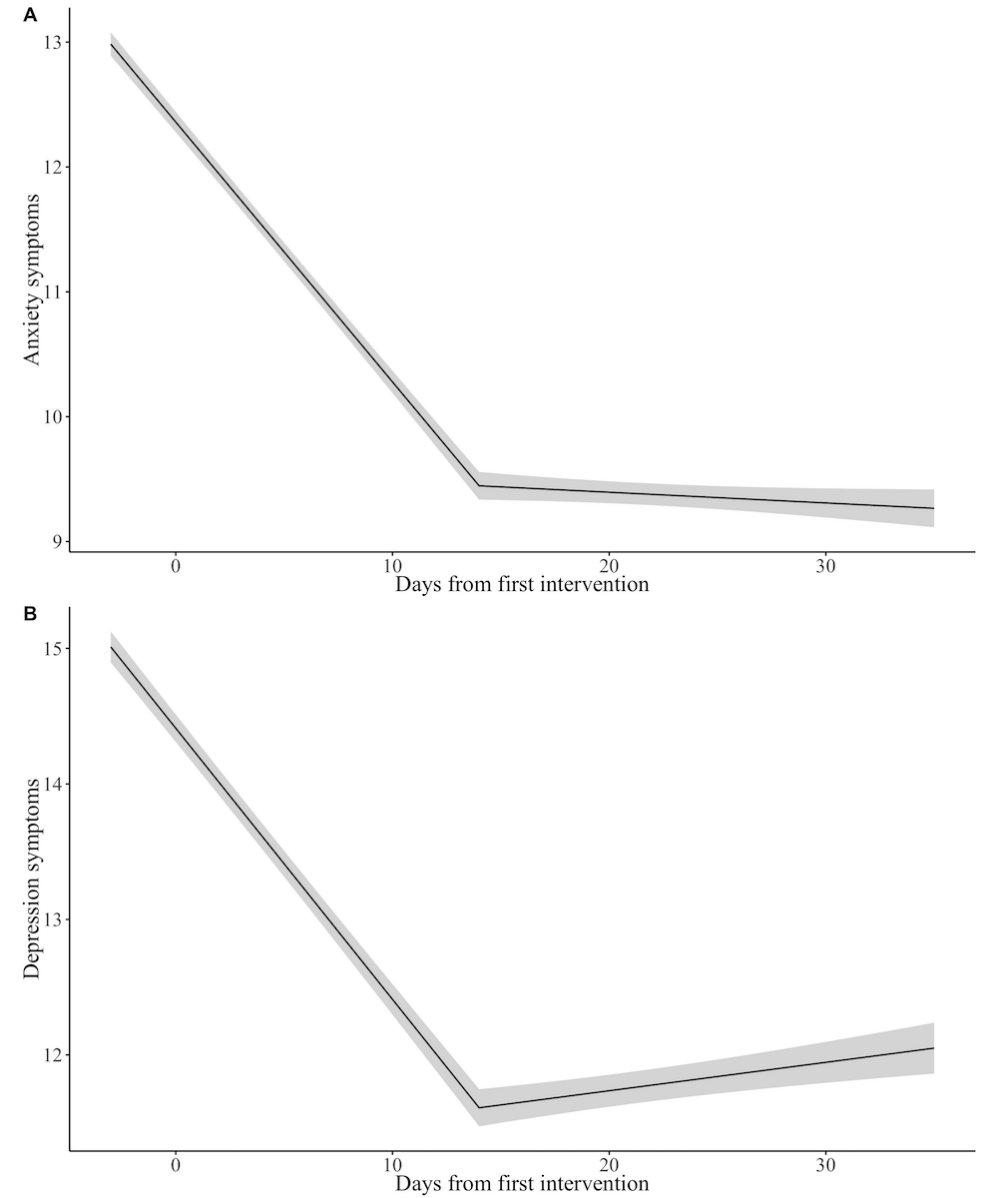
Symptom reduction over time in the full sample. The gray shaded region indicates bootstrapped SEs. Model details are described in the Results for Aim 2.

Results are displayed in Figure 2. Participants (N_participants_=4144; N_observations_=7093) experienced a significant reduction in anxiety symptoms from T0 to T1 (*b*=–0.21; bootstrapped 95% CI –0.22 to –0.19; *P*<.001). From T1 to T2, there was no significant change in anxiety symptoms (*P*=.35). The conditional means (and bootstrapped SEs) at day 0, day 14, and day 28 were 12.36 (SE 0.08), 9.45 (SE 0.11), and 9.33 (SE 0.11), respectively. These differences equate to Cohen *d*s of 0.57 between day 0 and day 14, 0.60 between day 0 and day 28, and 0.02 between day 14 and day 28. When analyses were conducted only on participants who had completed at least two assessments (N_participants_=2117; N_observations_=5066) or all 3 assessments (N_participants_=827; N_observations_=2481), results were unchanged.

#### Depression

Results are displayed in Figure 2. Participants (N_participants_=3992; N_observations_=6685) experienced a significant reduction in depression symptoms from T0 to T1 (*b*=–0.20; bootstrapped 95% CI –0.22 to –0.18; *P*<.001). From T1 to T2, depression symptoms increased slightly (*b* =0.02; bootstrapped 95% CI 0.00-0.04; *P*=.05). The conditional means (and bootstrapped SEs) at day 0, day 14, and day 28 were 14.41 (SE 0.10), 11.61 (SE 0.14), and 11.90 (SE 0.13), respectively. These differences equate to a Cohen *d* of 0.46 between day 0 and day 14, 0.42 between day 0 and day 28, and 0.05 between day 14 and day 28. When analyses were conducted only on participants who had completed at least two assessments (N_participants_=1951; N_observations_=4644) or all 3 assessments (N_participants_=737; N_observations_ = 2211), results were unchanged with the exception that symptoms no longer significantly increased between T1 and T2 (*P*=.08 and *P*=.15, respectively).

### Aim 3: Cumulative Regulation Hypothesis

#### Preliminary Analyses

We first examined the probability that users would successfully regulate their emotion within a conversation with Youper. As described in the methods, we defined successful regulation as a conversation that started with a negative emotion and ended with either a negative emotion at a lower intensity or a positive emotion. Using a generalized linear model with logit link function and random intercepts for each participant and each preintervention discrete emotion (N_participants_=4120; N_observations_=32,885), we found that overall, participants were more likely to succeed in regulating their negative emotion than to fail (OR 4.82, bootstrapped 95% CI 3.89-5.99; *P*<.001).

#### Anxiety

**Figure 3:**
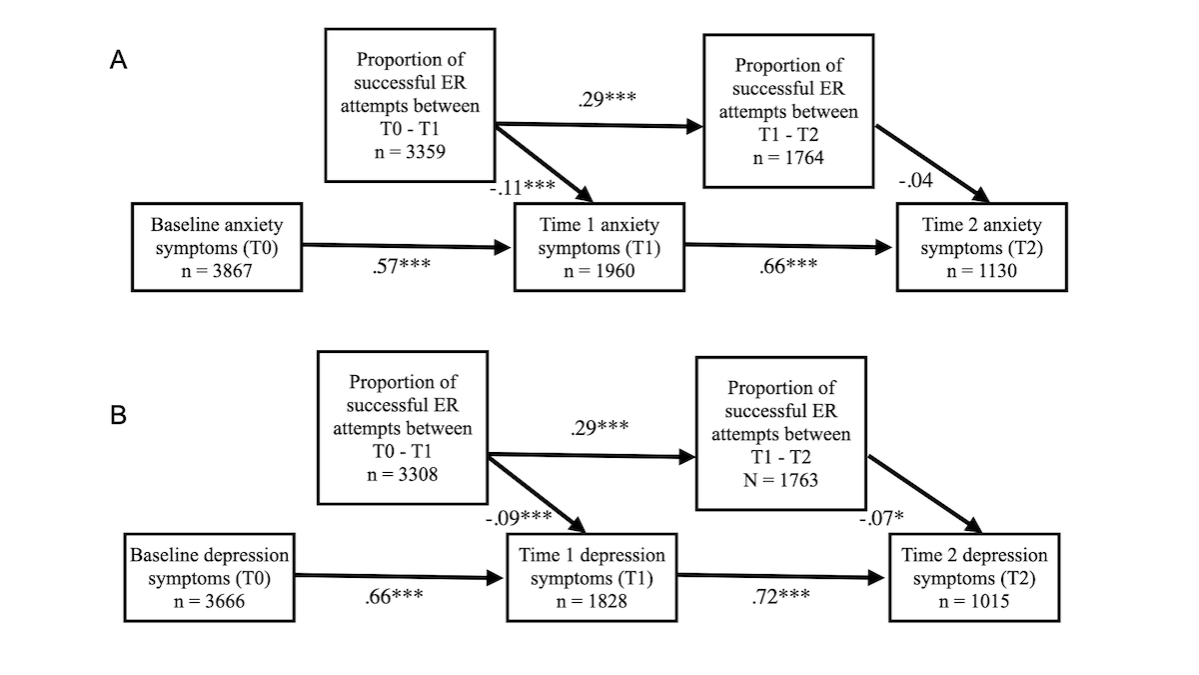
Path analysis diagram with standardized coefficients. This diagram shows the autoregressive and lagged relationships between the proportion of a user’s ER attempts that were successful out of their total regulation attempts and subsequent anxiety symptoms (A) or depression symptoms (B). ER: emotion regulation. ***P<.001, **P<.01, *P<.05. Exact *P* values are noted in the text.

To examine the effect of regulatory success within Youper sessions on anxiety symptoms, we fit a longitudinal path analysis model (N_participants_=4284; see Figure 3 for ns for each variable). The model had good fit characteristics as indicated by a significant chi-square value and standard fit statistics (*X^2^*_4_=60.84; *P*<.001; root mean square of approximation [RMSEA]=0.058; Tucker-Lewis index [TLI]=0.91; comparative fit index [CFI]=0.96; standardized root mean squared residual [SRMR]=0.046). This model estimated that for each 0.10 increase in the proportion of negative emotions that users successfully improved between T0 and T1, users reported a 0.20 point reduction on the GAD-7 anxiety measure at T1 (*P<*.001). The effect of the proportion of negative emotions that users improved between T1 and T2 did not significantly reduce subsequent GAD-7 scores at T2 (*P*=.19). See Figure 3a for standardized coefficients for all paths.

#### Depression

In order to examine the effect of emotion regulatory success on depression symptoms, we fit a similar longitudinal path analysis model (N_participants_=4228; see Figure 3 for ns for each variable). This model also had good fit characteristics as indicated by a significant chi-square value and standard fit statistics (*X^2^*_4_=50.93; *P*<.001; RMSEA=0.053; TLI=0.94; CFI=0.97; SRMR=0.041). For each 0.10 increase in the proportion of negative emotions that users successfully regulated between T0 and T1, they reported a 0.20 point reduction on the subsequent PHQ depression measure at T1 (*P*<.001). For every 0.10 increase in the proportion of negative emotions that users successfully regulated between T1 and T2, they reported a 0.13 point reduction in depression symptoms at T2 (*P*=.02). See Figure 3b for standardized coefficients for all paths.

#### Secondary Analyses

In addition to our primary hypotheses, we also conducted exploratory analyses of potential individual difference predictors of symptom reduction. In these analyses, we fit piecewise mixed effects models with a breakpoint at 14 days (time of T1 symptom assessment). We regressed the specified symptom assessment onto the interaction of the specified predictor and the number of days since the user subscribed to the app. We examined age, gender, whether the user was taking psychotropic medication, and whether the user was in therapy as individual difference predictors of symptom reduction.

#### Age and Gender

The interaction effects of time using Youper with age on anxiety (N_participants_=4143; N_observations_=7090) and depression (N_participants_=3991; N_observations_=6683) symptoms were not significant from T0 to T1 (*P_anxiety_*=.77; *P_depression_*=.39) or from T1 to T2 (*P_anxiety_*=.54; *P_depression_*=.43) in the piecewise regression models.

The interaction effects of time using Youper with gender on anxiety (N_participants_=4144; N_observations_=7093) and depression (N_participants_=3992; N_observations_=6685) symptoms were not significant from T0 to T1 (*P_anxiety-male_*=.74; *P_anxiety-nonbinary_*=.44; *P_anxiety-not-informed_*=.99; *P_depression-male_*=.16; *P_depression-nonbinary_*=.32; *P_depression-not-informed_*=.37) or from T1 to T2 (*P_anxiety-male_*=.40; *P_anxiety-nonbinary_*=.28; *P_anxiety-not-informed_*=.17; *P_depression-male_*=.89; *P_depression-non-binary_*=.70; *P_depression-not-informed_*=.25) in the piecewise regression models.

#### Number of Self-reported Diagnoses

The interaction effects of the number of self-reported diagnoses with time using Youper on anxiety symptoms (N_participants_=2679; N_observations_=4661) was not significant from T1 to T2 (*P*=.08), and not significant from T0 to T1 (*P*=.59).

There was a significant interaction effect of number of self-reported diagnoses with time using Youper on depression symptoms from T1 to T2 (N_participants_=2738; N_observations_=4589; *b***=**0.02; bootstrapped 95% CI 0.007-0.04; *P*=.006), but not from T0 to T1 (*P*=.78). This indicated that users with more diagnoses regressed modestly towards their baseline level of depression in the latter half of the treatment, whereas users with fewer diagnoses retained the treatment benefit.

#### Medication and Therapy

There were no significant interaction effects of taking prescribed medication with time using Youper on anxiety (N_participants_=2719; N_observations_=4733) or depression (N_participants_=2776; N_observations_=4654) symptoms from T0 to T1 (*P_anxiety_*=.32.; *P_depression_*=.72) or from T1 to T2 (*P_anxiety_*=.57; *P_depression_*=.66).

There were no significant interaction effects of receiving psychotherapy with time using Youper on anxiety (N_participants_=2719; N_observations_=4733) or depression (N_participants_=2776; N_observations_=4654) symptoms from T0 to T1 (*P_anxiety_*=.66; *P_depression_*=.87) or from T1 to T2 (*P_anxiety_*=.65; *P_depression_*=.52).

## Discussion

### Summary

The present study had 3 aims. First, we examined the acceptability of Youper AI therapy by assessing user ratings and retention metrics among subscribers. Second, we tested whether there were significant reductions in anxiety and depression symptoms. Third, we examined the cumulative regulation hypothesis, which predicts that the frequency of within-conversation emotion regulation success would predict symptom reduction.

Findings indicated that users were well retained and provided high ratings of Youper (median 5/5). As hypothesized, users showed significant reductions in symptoms in the first 2 weeks of using Youper with sustained improvements through 4 weeks from initial download. Finally, consistent with the cumulative regulation hypothesis, greater frequency of within-conversation emotion regulation successes significantly predicted greater reductions in anxiety and depression. Although no demographic predictors emerged, users with more self-reported diagnosed psychiatric conditions showed a slight return of depression symptoms between 2 and 4 weeks from first subscribing to Youper.

### Acceptability and Effectiveness

Because retention poses a significant challenge for entirely unguided treatment programs, our finding that 60.44% (2730/4517) of users continued to engage with the app in the second week and 42.66% (1927/4517) of users continued to engage with the app in the fourth week after initial download is promising. Although there are no clearly established metrics of retention for mobile apps, a recent paper examining retention among different mobile apps showed that Youper had the highest “stickiness” (measured by the ratio of active users to downloads in a given month) compared to any other treatment app for anxiety and depression [57]. Because Youper users experienced symptom improvements on average within the first 2 weeks of app use, with the present retention rate, it is likely that a large portion of users will stick with the app long enough to experience some positive effects. It is also notable that the median satisfaction rating given by users was 5 out of 5. Taken together, these findings indicate that Youper has great potential as a highly acceptable and adequately engaging digital treatment program.

Youper users showed a moderate effect size reduction for anxiety (*d*=0.57) and depression (*d*=0.46) within 2 weeks of starting app use. The reduction in anxiety symptoms was maintained through the 4-week period (day 0 to day 28: *d*=0.60). The reduction in depression symptoms was maintained through the 4-week period (day 0 to day 28: *d*=0.42) although depression increased slightly, but significantly, between weeks 2 and 4. These effect sizes are comparable to those found in RCTs of other commercially available mobile apps tested for a similar duration [9-11,58], suggesting that the AI therapy approach is viable for further testing in a randomized clinical trial. Youper users also had high success at regulating their negative emotions with each conversation. Given the low cost and potential for broad dissemination of Youper, these findings are particularly exciting, as they provide preliminary evidence of Youper’s effectiveness as an emotion regulation tool and a transdiagnostic treatment. It is important to note, however, that the final mean PHQ score of 11.9 still fell in the moderate severity range. Thus, as we begin to understand the mechanisms of the AI therapy approach and gain greater understanding of how to maximize user engagement, we are hopeful that effects on symptom reduction will continue to improve.

Youper’s symptom reduction, retention, and satisfaction ratings are notable because they were demonstrated in a real-world setting. Although highly controlled feasibility pilot trials allow determination of causal inference, these studies may not be generalizable to real-world settings and may fail to address issues of external relevance and dissemination [59]. Our analysis included a very large sample of Youper users who voluntarily downloaded and purchased the Youper program. Unlike in typical research settings, users were not recruited to participate or compensated for their assessments or for providing their feedback during their participation. Observed retention rates and symptom reduction therefore have already been shown in a real-world setting and population.

### Cumulative Regulation Hypothesis

The finding that cumulative within-session emotion regulation was strongly predictive of symptom reduction provides preliminary evidence for a potential mechanism of the AI therapy just-in-time intervention approach. Youper is theorized to enact its effects by enhancing emotion regulation skills via -in-time interventions. Thus, more effective emotion regulation sessions would indicate progress towards enhanced general emotion regulation skills and ultimately, symptom reduction. Therefore, it is promising that the effectiveness of the emotion regulation practice predicts the longer-term impacts of app use on symptom reduction. Although these results provide initial support for the theorized model underlying Youper’s treatment approach, randomization is critical for rigorously testing within-session emotion regulation as a mediator of symptom reduction.

### Predictors of Symptom Reduction

Interestingly, no demographic predictors of symptom reduction emerged. These findings are largely consistent with the existing literature where demographic features rarely predict symptom reduction [60-67]. These findings are promising, suggesting that digital treatment programs can be broadly disseminated with similar potential benefit across demographic groups. The number of comorbid diagnoses was a significant predictor of response such that users who reported more diagnosed mental health conditions showed a slight return of depression symptoms between 2 and 4 weeks from the first subscription date. These findings are consistent with prior literature showing poorer outcomes with greater comorbidity in depression treatment [68-70]. Users with more diagnosed conditions likely have a more severe clinical presentation, meaning that an entirely self-guided program may be less effective for this group. The finding that concurrent medication and therapy did not significantly impact symptom reduction suggests that the demonstrated effects of Youper on symptoms are unlikely to be explained by concurrent treatment, and that participating in other treatments alongside Youper does not hinder its effects.

### Limitations and Future Directions

Despite many strengths, our study had a few limitations. First, because these data were not collected as part of a research study, we did not have a control group, making it impossible to determine whether symptom reduction was simply due to the passage of time. However, given that effect sizes for symptom reduction that we found are comparable to those found in RCTs of other mobile app programs that showed significant differences between active treatment groups and wait list controls [9-11,58], it is unlikely that these effects can be explained by spontaneous remission. Second, because this was an observational study, we used the symptom data that were available to us, which included only self-report measures. Although we used validated measures, solely relying on self-report does not give a complete picture of the impact of Youper on clinical symptoms and overall functioning that could be more thoroughly assessed via clinical interviews. Third, this study included only 2 brief measures as outcomes: the PHQ and the GAD-7. Although these measures are widely used and show excellent psychometric properties, additional measures of anxiety, depression, and other purported outcome targets, such as quality of life and functioning, could help us better understand Youper’s effectiveness. Fourth, 47.84% (2161/4517) of Youper users were concurrently taking medication or engaging in therapy, meaning that it is possible symptom reduction resulted from participation in these other treatments rather than Youper (although concurrent treatment was not a significant moderator of symptom reduction). Finally, our emotion regulation measure was not designed to assess the magnitude of emotion regulation success, meaning that our metric included only success or failure with each conversation. These limitations should be addressed in future studies that include a control group, that assess symptoms using clinician-administered measures, that include a broader array of self-report measures, and that use more precise measures of emotion regulation success.

### Conclusions

This study provides preliminary evidence for Youper’s acceptability in a real-world setting that is unfettered by the constraints of highly controlled clinical trials. It also provides evidence of Youper’s effectiveness as an entirely unguided intervention for anxiety and depression. Finally, we demonstrated that Youper’s effects on symptom reduction may be explained by repeated within-session emotion regulation successes, providing preliminary support for the process by which a just-in-time intervention can be effective for the treatment of emotional disorders. Our results highlight the potential impact of Youper as a low-cost, light-touch, transdiagnostic intervention for anxiety and depression that can be broadly disseminated to improve mental health for millions of people around the world.

## Abbreviations

AI: artificial intelligence
CFI: comparative fit index
GAD-7: generalized anxiety disorder measure
PHQ-9: Patient Health Questionnaire-9
RCT: randomized controlled trial
RMSEA: root mean square of approximation
SRMR: standardized root mean squared residual
TLI: Tucker-Lewis index
